# Support, not blame: safe partner disclosure among women diagnosed with HIV late in pregnancy in South Africa and Uganda

**DOI:** 10.1186/s12981-024-00600-z

**Published:** 2024-03-13

**Authors:** Adelline Twimukye, Yussif Alhassan, Beate Ringwald, Thokozile Malaba, Landon Myer, Catriona Waitt, Mohammed Lamorde, Helen Reynolds, Saye Khoo, Miriam Taegtmeyer

**Affiliations:** 1 Infectious Diseases Institute, College of Health Sciences, Makerere University, Kampala, Uganda; 2 Department of International Public Health, Liverpool School of Tropical Medicine, Pembroke Place, Liverpool, L3 5QA UK; 3 Department of Clinical Sciences, Liverpool School of Tropical Medicine, Liverpool, UK; 4 Division of Epidemiology and Biostatistics, School of Public Health, University of Cape Town, Cape Town, South Africa; 5 Institute of Systems, Molecular and Integrative Biology, University of Liverpool, Liverpool, UK; 6 Tropical Infectious Disease Unit, Liverpool University Hospital Foundation Trust, Liverpool, UK

**Keywords:** HIV, Safe partner disclosure, Women living with HIV, HIV diagnosis late in pregnancy, Uganda, South Africa

## Abstract

**Background:**

HIV partner disclosure rates remain low among pregnant women living with HIV in many African countries despite potential benefits for women and their families. Partner disclosure can trigger negative responses like blame, violence, and separation. Women diagnosed with HIV late in pregnancy have limited time to prepare for partner disclosure. We sought to understand challenges around partner disclosure and non-disclosure faced by women diagnosed with HIV late in pregnancy in South Africa and Uganda and to explore pathways to safe partner disclosure.

**Methods:**

We conducted in-depth interviews and focus group discussions with pregnant women and lactating mothers living with HIV (*n* = 109), disaggregated by antenatal care (ANC) initiation before and after 20 weeks of gestation, male partners (*n* = 87), and health workers (*n* = 53). All participants were recruited from DolPHIN2 trial sites in Kampala (Uganda) and Gugulethu (South Africa). Topic guides explored barriers to partner disclosure, effects of non-disclosure, strategies for safe disclosure. Using the framework analysis approach, we coded and summarised data based on a socio-ecological model, topic guides, and emerging issues from the data. Data was analysed in NVivo software.

**Results:**

Our findings illustrate pregnant women who initiate ANC late experience many difficulties which are compounded by the late HIV diagnosis. Various individual, interpersonal, community, and health system factors complicate partner disclosure among these women. They postpone or decide against partner disclosure mainly for own and baby’s safety. Women experience stress and poor mental health because of non-disclosure while demonstrating agency and resilience. We found many similarities and some differences around preferred approaches to safe partner disclosure among female and male participants across countries. Women and male partners preferred healthcare workers to assist with disclosure by identifying the ‘right’ time to disclose, mentoring women to enhance their confidence and communication skills, and providing professional mediation for partner disclosure and couple testing. Increasing the number of counsellors and training them on safe partner disclosure was deemed necessary for strengthening local health services to improve safe partner disclosure.

**Conclusion:**

HIV diagnosis late in pregnancy amplifies existing difficulties among pregnant women. Late ANC initiation is an indicator for the likelihood that a pregnant woman is highly vulnerable and needs safeguarding. Respective health programmes should be prepared to offer women initiating ANC late in pregnancy additional support and referral to complementary programmes to achieve safe partner disclosure and good health.

**Supplementary Information:**

The online version contains supplementary material available at 10.1186/s12981-024-00600-z.

## Background

Safe partner disclosure of HIV status may advance the prevention of perinatal transmission of HIV alongside progress made through biomedical innovations [1]. Partner disclosure – the process of enlightening HIV status to a partner – is associated with improved antiretroviral therapy (ART) initiation, adherence, and retention in care among pregnant and postpartum women [2] and safe infant feeding practices [3, 4]. Further, women’s partner disclosure can boost partner relationships, through male partner’s involvement in pregnancy [5, 6], social support [7], HIV testing among male partners, and condom use among HIV sero-different partners [6, 8, 9] but evidence is inconsistent across settings [10, 11]. Non-disclosure, on the other hand, can cause worry and stress among pregnant women [12] leading to sub-optimal engagement in antenatal care (ANC) [13] and ART services [12, 14, 15], virologic non-suppression [16, 17] and infant feeding challenges [3].

Partner disclosure is difficult, more so during pregnancy [9, 12]. Rates of partner disclosure among pregnant women living with HIV range from 30 to 93% (pooled estimate: 64%) across Africa [18], also being lower for HIV-seropositive status compared to HIV-negative status [10, 19]. A smooth relationship, caring partner, and supportive health workers can facilitate partner disclosure [9, 20, 21], whereas fear of blame, anger, violence, abandonment, and/or loss of partner’s financial or emotional support deters partner disclosure [12, 21–23]. Although many partners react calmly and are supportive, partner disclosure can trigger negative outcomes, including anger and emotional, physical, or sexual intimate partner violence (IPV) [19, 24, 25].

Partner disclosure is a stated priority of perinatal HIV prevention programmes in many settings, including South Africa and Uganda, where data for this study were collected. In counselling sessions throughout pregnancy and postpartum periods, women living with HIV are encouraged to talk with partners about their HIV status. The World Health Organization (WHO) recommends assisted partner notification services to enhance safety of people living with HIV [26]. A facilitated couple counselling approach, combining couple counselling and counsellor facilitated partner disclosure, yielded high partner disclosure rates (81%) among sero-different couples in Uganda [27]. Couple testing and counselling alone can also increase social support for women living with HIV as shown in Malawi [28]. Family focused HIV care and treatment has been reported to facilitate HIV testing of male partners, men’s involvement in care of the family, and uptake of ART among men diagnosed with HIV in Côte d’Ivoire [29].

Research on HIV partner disclosure among women diagnosed with HIV later in pregnancy is scarce. While ANC in the first trimester of gestation is recommended, most pregnant women in sub-Saharan Africa initiate ANC in the second or third trimester (59%) [30], including in South Africa (53%) and Uganda (71%) [31, 32]. Women living with HIV who initiate ANC and ART late are at increased risk of disengaging from care [33] and failing to achieve viral suppression by delivery [34]. Women initiating ANC late and receiving HIV diagnosis later in pregnancy can be vulnerable (including due to poverty, relationship problems, and mistimed, unplanned, or unwanted pregnancies) [35, 36] and are faced with limited time to prepare for partner disclosure. Understanding the needs of these women could aid in providing additional support for safe partner disclosure. This study sought to understand challenges around partner disclosure and non-disclosure faced by women diagnosed with HIV late in pregnancy in South Africa and Uganda and to explore pathways to safe partner disclosure.

## Methods

### Study design and conceptual framework

This research was part of a qualitative cross-sectional study conducted alongside DolPHIN2, a randomised controlled trial (NCT03249181) of dolutegravir (DTG) use in pregnancy, which found the DTG-based regimen is safe, well tolerated, and results in rapid viral suppression when used later in pregnancy [1, 37]. We anticipated rapid viral suppression offered by DTG would encourage partner disclosure, and used qualitative methods to provide an in-depth understanding of the lived experiences of women living with HIV [38]. We explored a wide range of topics on prevention of perinatal transmission of HIV and use of dolutegravir in pregnancy. In this paper, we focus on data around safe partner disclosure of HIV. We employed a socio-ecological model [39, 40] to the data considering complex factors at individual, partner, relationship, family, community, health system, and societal levels influencing partner disclosure.

### Study settings, population, and recruitment

The study was conducted in Kampala, the capital city of Uganda, which accommodates about 1.6 million people, has an overall HIV prevalence of 7% [41]; and Gugulethu, a peri-urban township in Cape Town with approximately 100,000 residents, and an estimated female HIV prevalence rate of 19% [42]. Participants for the study were recruited from DolPHIN2 trial sites, the Infectious Diseases Institute HIV clinic and the Kasangati Health Centre, located in urban and peri-urban settings respectively in Kampala. In South Africa, participants were recruited from the Gugulethu Midwife Obstetric Unit, serving peri-urban low-income populations.

Participants were purposively selected from the same facilities and communities, including pregnant women living with HIV (up to six months postpartum), male partners, facility healthcare workers (HCWs) and community-based health workers (CHWs) (Table 1). Within the study settings, HCWs (e.g. ART case managers, clinicians, nurses, midwives) delivered HIV prevention and care while the CHWs (e.g. peer mothers, Village Health Teams) provided health education, referrals, follow-up, and case management through home visits. However, our analysis focused mainly on the experiences and views of women living with HIV participants who had initiated ANC after 20 weeks of gestation (late presenters) as they are most vulnerable and affected by disclosure challenges. Many of these had tested positive for HIV at their first ANC; most were enrolled in the DolPHIN2 trial. We utilised the views of the other participants, including women living with HIV who had initiated ANC within 20 weeks of gestation (early presenters), HCWs, CHWs, and male partners to triangulate and gain a well-rounded understanding of the study topic.

**Table 1:** Characteristics of study participants

IDIs with WLHIV	South Africa	Uganda
	*n* = 20	*n* = 22
Pregnant women	15	9
Recent mothers	5	13
**Age**		
18–24 years	3	2
> 24 years	17	20
**Education**		
Primary	6	10
Secondary or above	14	10
**Gestation at first ANC**		
< 20 weeks of gestation (early)	11	11
> 20 weeks of gestation (late)	9	11
**FGDs**	**n=9**	**n=6**
Women	40 (4 FGDs)	27 (3 FGDs)
Male partners	59 (5 FGDs)	28 (3 FGDs)
**Age**		
18–24 years	18	10
> 24 years	81	45
**IDIs with health workers**	**n=23**	**n=30**
Healthcare workers (HCWs)	15	15
Community health workers (CHWs)	8	15
**Total (n = 249)**	**n = 142**	**n = 107**

*Note* ANC = antenatal care; FGD = focus group discussion; IDI = in-depth interview; WLHIV = women living with HIV

Participants’ HIV status and gestational age at ANC booking were ascertained through self-reports. Trained research assistants (RAs) through the help of counsellors and ANC nurses identified women living with HIV from the study sites during their ANC, HIV care, or trial study visits and invited these women to participate in the qualitative study. Most male partner participants were recruited through female participants. Although not directly asked, it emerged from the focus group discussions (FGDs) that most male participants knew about their partners’ HIV-positive status. RAs approached potential respondents in person or by phone, informed them about the nature and purpose of the study, and obtained written informed consent prior to their participation.

### Data collection

Data were collected by RAs between August 2018 and March 2019 through 97 in-depth interviews (IDIs) and 15 FGDs, including 44 IDIs with women living with HIV and 8 FGDs with male partners (Table 1). IDIs provided participants with privacy to talk freely about this sensitive topic, and FGDs captured a wide range of views to triangulate data [43]. Topics explored included partner disclosure, effects of partner non-disclosure, strategies for coping with non-disclosure, and strategies for safe disclosure. Topic guides were piloted and revised based on relevant findings before data collection.

IDIs and FGDs were carried out in parallel, and emerging results from IDIs were explored further in FGDs. FGDs with 6–12 participants were conducted by two RAs, a facilitator and a note taker, trained in the study protocol, research ethics and with extensive local knowledge. Female RAs interviewed women participants. Data were collected in Xhosa in South Africa and Luganda in Uganda in safe and private locations within facilities (IDIs) and community centres (FGDs). IDIs and FGDs, lasting for approximately one hour, were audio-recorded.

### Data analysis

Independent transcriptionists in Uganda and South Africa transcribed audio recordings verbatim and translated them into English. We could not perform back-translation due to resource constraints; however, all transcripts were complemented by written notes and checked for accuracy and completeness. Data were analysed using the framework approach [44], aided by NVivo software [45]. We read all transcripts for recurrent ideas and developed codes inductively from the data. We used the socio-ecological model and topic guides to organise codes creating a common coding framework. The coding framework was reviewed, discussed, and agreed by the research team. We coded relevant text segments, and aggregated similar codes into themes, addressing the research question. A socio-ecological model was adapted to visualise themes. To ensure rigor and trustworthiness, we coded, compared, and discussed transcripts independently. Participant validation was achieved by presenting anonymised summary findings to a community advisory group in Uganda (including some participants) and integrated their feedback into the analysis. Emerging findings were regularly discussed by authors in meetings.

## Results

**Fig. 1:**
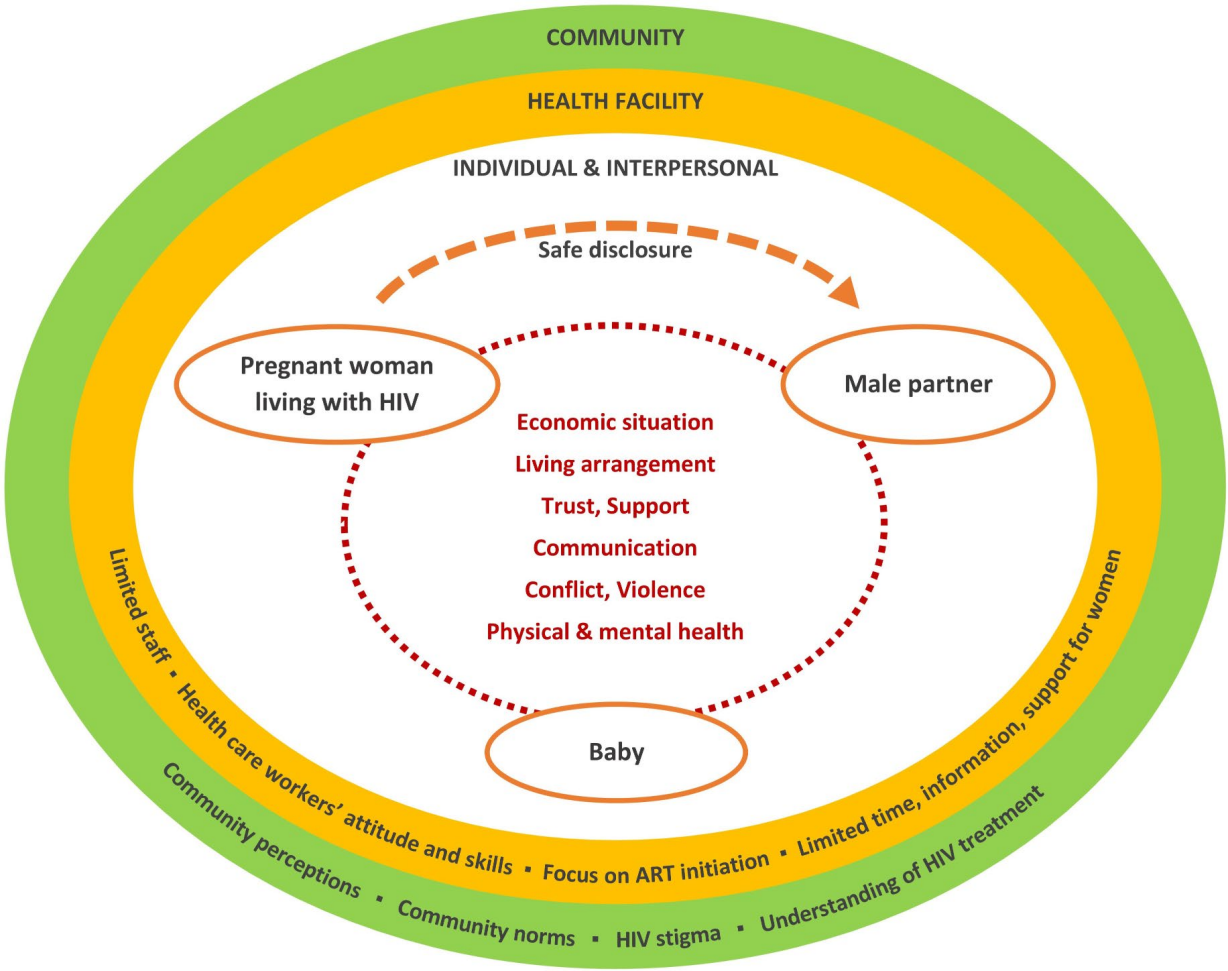
Socio-ecological framework of partner disclosure in late pregnancy. *Note* Interconnected factors at individual, interpersonal, health facility and community levels underpinning partner disclosure among women diagnosed with HIV late in pregnancy

Participant responses reveal multiple intersecting factors across individual, interpersonal, healthcare provider, and community levels that complicate partner disclosure of HIV status among women diagnosed later in pregnancy. Important themes of perceived linkage between partner non-disclosure later in pregnancy and women’s increased vulnerability to stigma, financial neglect, and violence from male partners as well as preferred approaches for partner disclosure are presented along with illustrative quotes. Figure 1 depicts the interrelated factors identified in a socio-ecological model of safe partner disclosure. We have focused mainly on the experiences of who presented late for ANC and reported non-disclosure, triangulated with perspectives from other participants (early ANC presenters, late ANC presenters who disclosed, male partners, and healthcare workers) to enhance understanding.

### Partner non-disclosure of HIV status in late pregnancy

Amongst the 20 women who were diagnosed with HIV late in pregnancy, only three said they had disclosed their HIV status to their partner within three months of diagnosis. These women were married, engaged in a viable income generating activity, and reported no major relationship issues like violence. Perceived need to adopt safer sex and to minimize risk of perinatal transmission motivated them to disclose. Being in a stable relationship aided partner disclosure. “*I was sure it was not me… I don’t sleep around so I was not feeling guilty and scared… to tell him”* (Woman, late ANC, disclosed, Uganda). Women experienced psychological relief, improved partner relationships and support for HIV treatment after partner disclosure.

It was fine. He also got tested after I told him, and he was negative…. he was very supportive, reminded me when to take my medicine (Woman, late ANC, disclosed, South Africa).

Many of the women who had not informed their partners about their HIV status said they disclosed to a female family member for psychological support.

Several male partners, whose women disclosed during late pregnancy, reported that disclosure was beneficial and facilitated support for the woman and protected the health of the rest of the family. Men who were already positive at the time of their partners diagnosis were more likely to accept and be sympathetic to their partners’ HIV status.

It’s a lady that brought me here…. saw that I was infected. So she was able to disclose to me when they found she was positive. I also told the one at home that I am infected, and I took her to our place and they found out she wasn’t infected. … we remind each other to take our medicine (Male FGD, Uganda).

However, disclosure also led to relationship breakdowns, as this male participant noted:

For me they got me a girl and they wanted me to marry her. … we met and she got pregnant. …she got drunk and she told me, ‘’ for me I am HIV positive… [but]I have been given medicine, and I use it well. And when you use the medicine well you cannot infect another person.” That was the end of our relationship. The person who got her for me called her and told her “You are stupid, if you go around telling everyone, they will run away you end up with none. …(Male FGD, Uganda).

Women’s reluctance to disclose to partners during late pregnancy was driven by individual, interpersonal, health facility, and community factors, as described below.

### Individual

One of the most frequently mentioned reasons was denial. Many women who were diagnosed with HIV late in pregnancy found it difficult to accept their HIV positive diagnosis because they had not experienced any signs and symptoms or any major health complications throughout their pregnancy. They preferred to wait a bit longer to ‘confirm’ the diagnosis or see if they could cope without disclosing, especially close to delivery.

When the doctor said I was positive, I did not believe it. Because I feel fine, my pregnancy has not given me any problem. I said let me wait, maybe it is a mistake (Woman, late ANC, non-disclosed, South Africa).

Late ANC attendees commonly experienced guilt for not seeking ANC earlier and thereby increasing perinatal HIV transmission risk. Women often believed that delayed diagnosis automatically meant their baby would acquire HIV, leading to psychological barriers to disclosure.

When I consider myself pregnant with HIV condition, I feel very bad! The baby could be infected. I just say, it is better to die with my AIDS-related illness than disclosing… (Woman, late ANC, non-disclosed, Uganda).

Poverty and financial dependency were other frequently cited reasons. Many women feared relationship problems and loss of financial support upon HIV disclosure to their partner, particularly in unstable relationships. This fear intensified during late pregnancy and early breastfeeding when women required more financial assistance and had limited capacity for paid work.

If I tell him, he may refuse to take care of my child…. As soon as he gets to know I am HIV+, he will run away and leave me (Woman, late ANC, non-disclosed, Uganda).

As a result, several late presenters preferred to defer partner disclosure until after childbirth.

So, what pregnant mothers tell us is, ‘Doctor, you wait for me to deliver because I do not have financial support, after delivery, I will inform him… (CHW, Uganda).

Partner disclosure was widely noted to be easier for women who were gainfully employed and able to sustain themselves financially. In addition, women felt they lacked essential communication skills.

I do not know how to disclose to my partner, how to talk to him so he does not get upset. It is something I am still looking, hoping the doctors will teach me (Women FGD, Uganda).

HIV issues were deemed particularly sensitive during pregnancy and thus require extreme care when engaging in conversations on such issues.

### Interpersonal relationships

Trust played a crucial role in disclosing HIV status to a partner during late pregnancy. It fostered open communication, confidentiality, and reassurance against accusations of infidelity.

It depends on how much we trusted each other. I was free to talk to my husband because I trusted him, and he also trusted me. I knew he would keep it a secret and not talk to anyone about my HIV… or accuse me (Women FGD, South Africa).

Across both countries, many women who initiated ANC late did not live with their partner and felt less urgency to disclose as they could easily keep their status a secret. Such separate living arrangement undermined communication and trust between partners which triggered violence when one of the partners was diagnosed with HIV.

Relationship issues undermined women’s ability to disclose to their partner during late pregnancy. *“Already we were having issues, always fighting. So, I felt it makes matters worse if I told him”* (Woman, late ANC, non-disclosed, Uganda). Women experiencing relationship challenges feared their partner’s response to disclosure, especially potential physical, emotional, and sexual abuse. “*I am afraid of telling him because maybe he might beat me up or kill me… judging by his character”* (Women FGD, South Africa). Late pregnancy HIV diagnosis heightened vulnerability to partner violence due to increased concerns of perinatal transmission and doubts about paternity, as illustrated by this male partner,

… six months into the pregnancy you will have spent a lot. Then you are told, ‘she is positive.’ You start to have doubts that the baby might not be yours, you feel cheated… (Men’s FGD, South Africa).

Pregnant women in South Africa reported partners’ alcohol use triggered aggression and impatience, which hindered disclosure. *“… I don’t have the guts… it is not easy… especially because he is always drunk…”* (Women FGD, South Africa). Women felt increased vulnerability to the consequences of violence for themselves and the unborn baby later in pregnancy, and often chose to not disclose to protect the unborn baby.

### Community

With HIV widely associated with promiscuity and infidelity, most women adopted non-disclosure as a strategy to mitigate stigma. Men’s response to their partner’s HIV diagnosis during pregnancy was influenced by fear of community gossip and stigma. When HIV diagnosis occurred later in pregnancy, couples are presented with limited option to deal with and avert the stigma, including potential termination of the pregnancy. In Uganda, couples were further constrained by the illegality of abortion in the country.

You feel people … will laugh at you, they will say “look his wife is positive but he is not, maybe she has been cheating” … it is not a good feeling. If she [tested positive] when they pregnancy is still small you can terminate but after 20 weeks it is too late… (Men’s FGD, Uganda).

Moreover, limited community understanding of ART effectiveness, including newer drugs like dolutegravir, undermined partner disclosure.

When someone sees you giving birth when you are HIV positive, they think you are stupid… because you will give birth to a child having HIV… (Women FGD, Uganda).

Stigma towards pregnant women living with HIV and blame for perinatal transmission further hampered disclosure, causing anxiety and affecting confidence in informing partners.

### Health facility level

Women found health care providers’ support toward partner disclosure after HIV diagnosis insufficient, especially when diagnosed late in pregnancy. Women, who initiated ANC late in pregnancy, were rushed through pre- and post-test counselling for ART initiation and felt overwhelmed with information. Time with and information from counsellors were inadequate.

Counsellors did not give us enough information because of lack of time, the clients were many yet they [counsellors] were few (Women FGD, Uganda).

Women needed time to process the information, evaluate potential effects of partner disclosure and therefore felt ill-prepared for partner disclosure.

I need time to think about how to discuss HIV topic with my partner. When am ready I can ask him… Basing on his response I may be able to disclose or give it time (Woman, late ANC, non-disclosed, Uganda).

Shortage of counsellors and insufficient skills restrained providers from effectively engaging with pregnant women living with HIV for disclosure readiness. HCWs, responsible for ANC and HIV services, required extra time for late first ANC visits due to numerous tests and checks within a limited timeframe. *“Some counsellors rush through the session with pregnant mothers due to lack of time…”* (HCW, Uganda). Further, some HCWs’ lack of compassion and negative attitude towards late ANC seekers hindered education and support for partner disclosure.

I fear being shouted at the clinic. In fact, when I booked late, I got shouted at, it is hard to learn anything so that you can disclose (Woman, late ANC, non-disclosed, South Africa).

Lack of trust in HCWs impeded disclosure, as pregnant women living with HIV feared their personal information wouldn’t be kept confidential. In Uganda, this lack of trust undermined assisted partner notification due to concerns about disclosing information about multiple partners.

Sometimes women fear disclosing to us (health workers) right away when they present for ANC, because they fear we may disclose to their partners… (HCW, Uganda).

### Effects of partner non-disclosure in late pregnancy

Many women concealed their medication due to non-disclosure, which could lead to disengagement and sub-optimal adherence.

The time I decided to swallow the drugs can approach when my husband is still at home, so I hide it from him and wait for him to leave… at times I find myself going past the time (Late ANC, non-disclosed, Uganda).

Dolutegravir, with its small size and low pill burden, allowed discreet concealment of HIV status and treatment until disclosure readiness. Women reported experiencing cognitive dissonance from the need to inform their partner to protect them and the baby on the one hand, and a feeling of unpreparedness and concern about their safety if they disclosed to their partner on the other. Many others reported experiencing anxiety, despair, anger, confusion, regret, guilt, and a negative outlook on life. Such negative psychological effects often complicated pill taking and clinic attendance among the women.

### Perceptions of approaches for safe partner disclosure

We found disclosure preferences among female and male participants overlapped overall, with some country-specific differences.

### Timing of disclosure

The timing of partner disclosure was important to both women and men. Similar to women’s reports (shown above), men felt their partner’s disclosure of HIV during late pregnancy would be stressful.

It will be difficult when she is pregnant and she says she is HIV positive… that is something else… that would be hard for me (Men FGD, South Africa).

Men’s expectations placed the responsibility on women to determine the timing and manner of disclosure, increasing women’s stress. Men emphasised the importance of polite communication about HIV to prevent anger and foster understanding. In South Africa, most men preferred immediate disclosure upon HIV diagnosis.

I think the sooner the better. I think you should disclose it immediately… so he can also go test. You need to handle the situation before it gets worse. You might be hiding it from him and maybe he too has it (HIV) (Men FGD, South Africa).

Other men, mainly from Uganda, did not want women to disclose during a stressful situation like financial problems.

When someone is stressed and there is a lot of poverty and then one brings such news; you can see as though you are both going to die tomorrow from AIDS-related illness; but it needs to be built on slowly (Men FGD, Uganda).

Some men and women preferred women diagnosed with HIV late in pregnancy to defer disclosure until after giving birth. “*It’s good to wait and tell him after delivery… you can manage if he leaves you or stop giving you money”* (Woman, late ANC, non-disclosed, South Africa). However, participants recognized that prolonged non-disclosure could foster mistrust and complicate future disclosure.

The longer you leave it the harder it gets. When you leave it too late, he will suspect that you are the source… (Women FGD, South Africa).

Participants acknowledged that the optimal situation for safe disclosure varied among families, and suggested HCWs assist couples in identifying the ‘right’ moment and provide ongoing guidance to women diagnosed late in pregnancy. Stepwise disclosure and collaborative preparation with HCWs were preferred by most women.

### Professional assisted disclosure and couple testing

Women and men in South Africa and Uganda preferred health worker-initiated or mediated disclosure over self-disclosure. HCW involvement provided counselling support and reduced blame and conflict.

If am scared to tell him myself, I bring him to the hospital then the basawo (healthcare workers) counsel him … so he can understand (Woman, early ANC, disclosed, Uganda).

Some women preferred couple testing and receiving test results simultaneously with their partners, along with couple counselling.

The easiest way is to get everyone to test, it becomes easy when couples go together to the testing stations and test together (Woman, late ANC, non-disclosed, South Africa).

However, men’s reluctance to visit ANC posed a challenge for this approach, although some men welcomed couple counselling.

When she is tested…the government should provide her with a letter stating that I should come to the clinic with her… so that we are both tested by a professional who can provide us with advice (Men FGD, South Africa).

A key challenge noted with HCW-mediated disclosure was the lack of trust between HCWs and individuals not ready to disclose. Moreover, limited capacity in terms of time and skills undermined HCWs’ ability to intervene, with some facilities lacking professional counsellors. Insufficient services for addressing IPV and mental health were also reported as barriers to professional-mediated disclosure in both settings.

Some women proposed home visits for testing and counselling, but concerns about unintended disclosure and stigma arose, particularly in extended or polygamous families lacking privacy for confidential testing. Prior notification and integrating home testing into general health promotion community outreaches could mitigate stigma.

### Empowerment for safe disclosure

Women in both countries highlighted the importance of individual and economic empowerment alongside safe partner disclosure interventions. They urgently needed skills in communication, timing, and strategies for disclosure, and sought mentoring and coaching.

I request you that you bring us those trainings, if you face such a situation then you know how to go about it (Woman, late ANC, non-disclosed, Uganda).

In Uganda, women identified the potential role of peers; other mothers living with HIV could support women, who were newly diagnosed, to identify the right time and approach to disclose. Income-generating activities were also desired to enhance financial independence.

I suggest women have their own source of income to take care of self in case man leaves after disclosure (Women FGD, Uganda).

## Discussion

Our findings indicate HIV diagnosis late in pregnancy complicates partner disclosure as it exacerbates women’s vulnerabilities that contribute to delayed ANC initiation. Whilst physical, mental, economic, and social wellbeing of pregnant women living with HIV, her partner, and their child are mutually connected, wider community and health system factors influence individual and interpersonal issues, all of which affect partner disclosure in late pregnancy. Denial of HIV, poverty, and poor mental health at the individual level are often accompanied by mistrust, conflict, lack of communication within partnerships. Guilt over late ANC initiation and worries about vertical HIV transmission during late diagnosis and being ‘the source of HIV’ create psychological barriers to partner disclosure. Common HIV misconceptions and stigma as well as HCWs attitude, priority, and lack of time further discouraged women from partner disclosure. Our findings resonate with evidence highlighting the complex and vast challenges that people living with HIV must navigate within their lives, relationships, and the health system to initiate and maintain ART [46].

The findings echo previous studies in South Africa and Uganda stressing fear of IPV as a driver of pregnant women’s reluctance to disclose own HIV status to their partner [12, 21–23]. Fear of IPV following partner disclosure is common among women who have previously experienced partnership conflicts and IPV [12, 22], sometimes in the context of partner’s alcohol use, financial dependency and poverty [12, 21], especially late in pregnancy. The nexus of late ANC booking, HIV and IPV is often underpinned by a clustering of social and economic vulnerabilities, discouraging partner disclosure [12, 21], complicating engagement in care, and potentially impeding the health of the woman and the baby.

Women who are diagnosed with HIV late in pregnancy need increased support around IPV within routine ANC and ART counselling processes. Counselling is an integral part of the ART initiation process in Uganda and South Africa, benefits can be limited when reduced to providing women with treatment and adherence information [47]. IPV screening in healthcare settings is likely to increase identification of women undergoing IPV [48]. Various screening tools like the violence against women tool [49] and conflict-tactics scales [50] have been validated. However, IPV screening, counselling, and safety planning as standalone without additional advocacy or therapeutic interventions are ineffective in reducing IPV [48, 51]. Interventions engaging multiple stakeholders and addressing multiple drivers at different levels are more likely to be effective [51].

Voices of women and men in this research highlight expectations, pressure and stress are high during the late stages of pregnancy. An HIV diagnosis late in pregnancy exacerbates the mental challenges, partly due to perceived high chances of perinatal transmission. HCWs, often prioritising physical health like HIV viral load, need to recognise mental health challenges affecting pregnant women living with HIV to inform counselling and other support plans. Dolutegravir, with its benefits of rapid viral reduction, has the potential to provide reassurance [52] and improve the mental health resilience of women when diagnosed with HIV late in pregnancy [53]. This could in turn encourage women to disclose HIV-positive status to their partner. HCWs should raise awareness on the drug’s effectiveness during counselling with women who are diagnosed late in pregnancy to maximise potential positive impact on women’s mental health and disclosure benefits for HIV care and prevention of HIV transmission.

Women in our study proposed a gradual approach to partner disclosure and additional support from counsellors to identify the right time and approach to disclose safely. This preference may run counter to the HIV Prevention and Control Act 2014 in Uganda providing for mandatory HIV testing and permitting medical providers to disclose a client’s HIV status to their partner [54]. While this legislation may have increased partner disclosure [55], its effects on the safety and dignity of women would merit systematic examination. A gradual approach to disclosure might result in procrastination and delayed disclosure, which is not ideal for improving health outcomes. However, it may be necessary in contexts where HIV is highly stigmatised, with women often blamed as the source of infection. As the data in this study revealed, inappropriate disclosure could heighten the risk of IPV for women and socioeconomic vulnerability. The gradual approach to disclosure prioritizes the safety and well-being of pregnant women while also ensuring men’s protection and their right to know. This approach is grounded in a human rights perspective.

In both countries, community misperceptions linking women’s HIV diagnosis during pregnancy with women’s promiscuity on one hand and automatic perinatal transmission on the other hand were particularly pertinent in promoting stigma and discouraging partner disclosure. Despite interventions to reduce HIV stigma in Uganda and South Africa [56, 57], greater emphasis needs to be placed on the benefits of ART in reducing perinatal transmission to improve the situation of pregnant women living with HIV. Health information and services should be accompanied by strategies for social empowerment, including improved partner communication [58], and economic empowerment like participation in income generation activities to address the structural barriers underpinning both late ANC engagement and HIV non-disclosure in pregnancy [59–61].

**Fig. 2:**
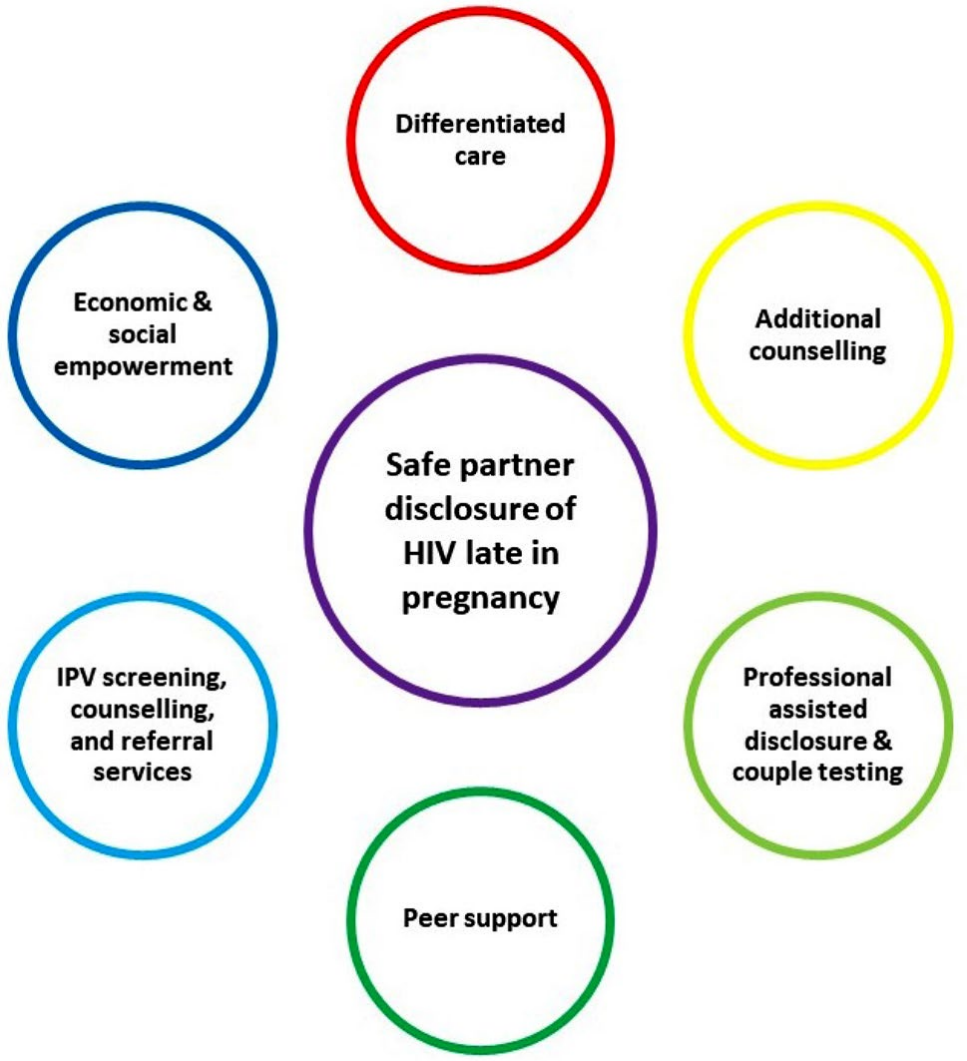
Recommended care and support for women diagnosed with HIV late in pregnancy

In summary, women who seek ANC late in pregnancy need a differentiated approach that recognises their unique, often difficult, circumstances, which make them delay ANC. Women who are diagnosed with HIV late in pregnancy need extra time, counselling and compassion. Figure 2 illustrates the recommended care and support packages that women diagnosed with HIV late in pregnancy for safe partner disclosure and, moreover, for her own health and well-being.

## Limitations

Our study had several limitations. Data were collected in two peri urban settings and was cross sectional, which limited disclosure perspectives over time. Nevertheless, we obtained relevant information about barriers to disclosure, effect of non-disclosure and mechanisms to facilitate partner disclosure. The study is based on self-reports and asked about women’s experiences of disclosure to their partners and therefore may be subject to recall bias. There is also a potential for social desirability bias and under-reporting of barriers to disclosure among respondents hesitant to share their true beliefs within group settings. Male participants found it challenging to discuss issues of disclosure in the group setting and individual IDIs may be well suited for exploring this in future. Further, our facility-based recruitment strategy meant we did not include the perspective of women living with HIV who were less engaged with care who might be faced with unique barriers to partner disclosure not covered by our data.

## Conclusions

Being diagnosed with HIV late in pregnancy increases women’s vulnerability and challenges, which make partner disclosure more difficult. Late ANC booking is an indicator for the likelihood that a pregnant woman may be highly vulnerable, needs additional support, and may require safeguarding. Differentiated care may help HCWs provide additional support to pregnant women who are diagnosed with HIV late in pregnancy, including ‘right’ timing and approach to assisted partner disclosure. Respective health programmes should be prepared to offer women initiating ANC late in pregnancy additional support and referral to complementary programmes to achieve safe partner disclosure and good health.

### Electronic supplementary material

Below is the link to the electronic supplementary material.

Supplementary Material 1

## Data Availability

Data are available from the corresponding author on request.

## Abbreviations

ANC: Antenatal care
ART: Antiretroviral therapy
CHW: Community Health Workers
FGD: Focus group discussion
HCW: Healthcare workers
HIV: Human immunodeficiency virus
IDI: In-depth interview
IPV: Intimate partner violence
RA: Research assistant
