# Supplementary material for: Support, not blame: safe partner disclosure among women diagnosed with HIV late in pregnancy in South Africa and Uganda
Source: AIDS Res Ther. 2024 Mar 13;21:14. doi: 10.1186/s12981-024-00600-z (PMC10938717; doi:10.1186/s12981-024-00600-z)
Supplement: Supplementary file 1 — Supplementary Material 1 [file 12981_2024_600_MOESM1_ESM.docx]

**Topic guides**

1. **In-depth interview guide for HIV positive pregnant and recent mothers**
2. Tell me about your experience of receiving your HIV positive diagnosis during pregnancy.

- Probe: how and where was the test carried out?
- Probe: how did you feel about the positive result? What were your concerns?
- Probe: would you have felt different if you found out outside pregnancy?

1. How easy or difficult was it for you to inform your partner and other family members about your HIV positive status?

- Probe: who did you tell about your diagnosis? When? how? And reasons for disclosing.
- Probe: Who did you not disclose to? Why? Are you planning to disclose to them at some point? When? Why?
- Probe challenges face disclosing to partner?

1. How did diagnosing (for HIV) in pregnancy affected your ability to disclose to your partner?

- Probe reasons for this
- Probe: would it have been easier for you to disclose if you were diagnosed outside of pregnancy?

1. [PARTICIPANTS WHO HAVE DISCLOSED] what factors helped you to be able to disclose to your partner?
2. What support did you receive from healthcare workers to help you disclose your status to your partner?

- Probe: how did you feel about the support?
- Probe: what would you like to be changed about the support given

1. How did diagnosing late in pregnancy affect your ability to disclose?

- Probe: would it have been easier for you to disclose to your partner if you were diagnosed earlier in pregnancy or when not pregnant?

1. In what ways has not disclosing your status to your partner affected you?

- Probe effects on treatment adherence
- Probe effect on relationship
- Probe effects on mental health and general wellbeing

1. What coping mechanisms did you employ to deal with non-disclosure to your partner?
2. How would you like partner disclosure to be done?

- Probe: what will make it easier for you to disclose during pregnancy/late pregnancy?

1. What additional support would help women who are diagnosed with HIV late in pregnancy to be able to disclose to their partner?
2. **FGD guide for women living with HIV**

1. How do people in your community perceive women who are HIV positive and are pregnant?

- Probe stigma and discrimination related to HIV in pregnancy.
- Probe perception of HIV diagnosis late in pregnancy.

1. What challenges do women face when they are diagnosed with HIV in pregnancy?

- Probe challenges faced when diagnosis occur late in pregnancy.

1. How easy or difficult is it for pregnant women to disclose their HIV positive status to their partner?

- Probe reasons for non-disclosure during pregnancy
- Probe motivations for disclosure during pregnancy

1. How does diagnosing (for HIV) late in pregnancy affect women’s ability to disclose to their partner?

- Probe: would it be easier for women to disclose to their partner if diagnosed earlier in pregnancy or when not pregnant?

1. How does partner non-disclosure affect the HIV positive pregnant woman?

- Probe effects on treatment adherence
- Probe effect on relationship
- Probe effects on mental health and general wellbeing

1. What coping mechanisms do pregnant women employ to deal with non-disclosure (to their partner)?
2. What will make it easier for women who are diagnosed with HIV later in pregnancy to be able to disclose their status to their partner?
3. What additional support from healthcare professionals will help women diagnosed with HIV late in pregnancy to be able to disclose to their partner?
4. **In-depth interview guide for facility and community health workers**
5. What reasons do women provide for not disclosing their HIV positive status to their partners during pregnancy?
6. How does HIV diagnosis later in pregnancy affect women’s ability to disclose to their partners?
7. What kind of support do you provide women diagnosed in pregnancy to be able to disclose?

- Probe specific interventions for women diagnosed late in pregnancy.

1. What challenges do you face as health workers in providing support to women to enable them to disclose their HIV status to partners?
2. How do you think women may be best supported to disclose to their partners during pregnancy?

- Probe specific support for women diagnosed late in pregnancy.

1. **FGD guide for male partners/participants**
2. How do men perceive spouses who are diagnosed positive for HIV during pregnancy?

- Probe perceptions of HIV diagnosis late in pregnancy.
- Probe how they (would) feel about their spouses testing positive for HIV in pregnancy.
- Probe if the stage of pregnancy when diagnosis is made would make any difference in terms of how they feel and perceive of the diagnosis.

1. Why do you think are the reasons why women find it difficult to disclose their HIV status to their partners?

- Probe challenges faced when diagnosis occur in pregnancy.
- Probe challenges faced when diagnosis occur late in pregnancy.
- Probe: what role do you think men have to play in this problem?

1. How does diagnosing (for HIV) late in pregnancy affect women’s ability to disclose to their partner?

- Probe: would it be easier for women to disclose to their partner if diagnosed earlier in pregnancy or when not pregnant?

1. How does partner non-disclosure affect the HIV positive pregnant woman?

- Probe effects on treatment adherence
- Probe effect on relationship
- Probe effects on mental health and general wellbeing

1. How would you like your spouses to disclose their HIV status to you if they are found to be positive during pregnancy?
2. What will make it easier for women who are diagnosed with HIV later in pregnancy to be able to disclose their status to their partner?
3. What additional support from healthcare professionals will facilitate partner disclosure of HIV positive status late in pregnancy?
